# Claudins: New Players in Human Fertility and Reproductive System Cancers

**DOI:** 10.3390/cancers12030711

**Published:** 2020-03-18

**Authors:** Marta Justyna Kozieł, Karolina Kowalska, Agnieszka Wanda Piastowska-Ciesielska

**Affiliations:** Medical University of Lodz, Department of Cell Culture and Genomic Analysis, Lodz 90-752, Poland; marta.koziel@umed.lodz.pl (M.J.K.); karolina.kowalska1@umed.lodz.pl (K.K.)

**Keywords:** claudins, tight junction, fertility, cancer

## Abstract

Claudins are major integral proteins of tight junctions (TJs), the apical cell–cell adhesions that enable maintaining polarity of epithelial cells, their differentiation, and cell signaling. A number of studies have indicated that claudins might play a crucial role in both physiology and pathogenesis. Their tissue-specific expression was originally linked to the development of different types of cancer and triggered a hope to use them as diagnostic or prognostic markers. However, it seems that their expression is more complex than that, and undoubtedly, claudins participate in one of the most important molecular events in cells. This review summarizes the recent research evaluating the role of claudins in fertility and the most common endocrine-dependent cancers in the reproductive system and highlights the crucial role of claudins both in human fertility and the most common cancers.

## 1. Introduction

In most living organisms, a basic function of epithelial and endothelial cells is to protect organs from their surroundings and maintain homeostasis [1]. The protective barrier of cells is provided by tight junctions (TJs), adherence junctions (AJs), and desmosomes [1]. TJs, known as occluding junction or zonula occludens, is a multiprotein complex that maintains cell barriers but also enables intercellular communication and transport between cells [2]. TJs most often occur in the apical part of epithelial cells. Occludin, claudin, and junction adhesion molecule (JAM) are three major transmembrane proteins of TJs.

**Figure 1:**
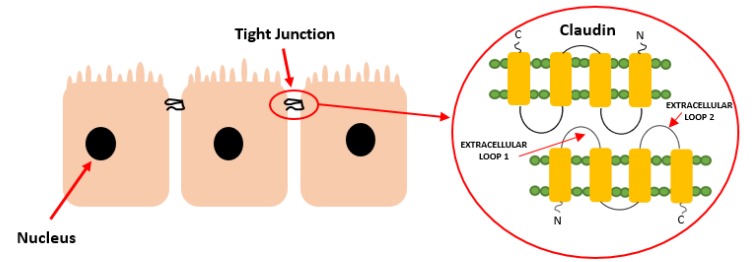
Structure of claudins, as a part of a tight junction.

Claudins are small, 20–34 kDa proteins sharing a common overall structure which possess four transmembrane domains, N-terminal and C-terminal ends located in the cytoplasm, and two extracellular loops [3] (Figure 1). The first extracellular loop is involved in the regulation of paracellular charge and ion selectivity. The second extracellular loop participates in adhesion of claudin–claudin interaction and works as a receptor for bacterial toxin [4,5]. The C-terminal end is the most heterogenic both in the sequence and length among claudins. The length of the C-terminal end in mammals is between 22 and 55 amino acids, whereas the N-terminal end is about seven amino acids [6]. The C-terminal end contains a PDZ-domain-binding motif, as well as a site of many post-translational modifications, and thus differs in sequence. All claudins, with the exception of claudin-12, have a PDZ motif which binds to other TJs cytoplasmic protein such as zonula occludin 1 (ZO-1) and allows interaction with other proteins [5]. This motif is also responsible for post-translational modifications, which might affect the localization and function of these proteins. For example, phosphorylation of claudin-1 by mitogen-activated protein kinase (MAPK) promotes barrier function, whereas protein kinase A (PKA) phosphorylation of claudin-16 exacerbates Mg^2+^ transport [7].

The claudins family consists of about 24 members whose basic function is to regulate pore sizes between polarized cells and regulate ions and neutral solutes transport directly [2]. These integral membrane proteins regulate the flow of ions and nutrients and are involved in maintaining homeostasis and communication between cells [8]. Claudins differ in their selectivity for ions, and might be cation-selective (Na^+^, K^+^, Ca^2+^) like claudin-2 or -15, or anion-selective (Cl^−^) like claudin-4, -7, and -10. Thus, the differences between barrier function are mostly associated with its selective permeability defined by claudins and its formed pores.

Claudins are encoded by CLDN genes that have up to four introns and, thus, are usually small genes. Part of them possess a similar sequence and are located closely, such *CLDN6* and *CLDN9* on chromosome 16, which suggests that they might have been generated by duplication and, in consequence, their regulation might be linked [9]. However, claudins differ in sequence similarity and based on that are divided into two groups: classic and non-classic. Classic claudins possess a high homology and include fourteen claudins, whereas left ten claudins share the least sequence homology [2,6,10] (Table 1). The expression pattern of claudins differs among tissues, and most of them express a number of claudins that might form different TJs and interact with other TJs proteins [11]. For example, claudin-7 is highly expressed in different parts of small and large intestine whilst claudin-6 is not observed in this tissue [12]. However, similar complex patterns of claudins expression are found in, e.g., kidney or prostate tissues [13].

The role of claudins in maintaining cell barrier as well as small ions and neutral solutes selective transport is crucial: experimental claudins knockout models lacking *CLDN1* and *CLDN5* are lethal [14,15]. Overexpression of claudin-1, claudin-4, claudin-5, and claudin-7 increase transepithelial resistance (TER), whereas claudin-2 and claudin-10 present contradictory effect in cultured in vitro epithelial cells [16]. Mutations in claudin genes are associated with many diseases: sclerosing cholangitis, hypomagnesemia, or ichthyosis. Claudins also play a crucial role in enterotoxin and viruses infections (hepatitis C virus, HCV) [17]. The disruption of TJs and the induction of epithelial to mesenchymal transition (EMT) in cancerogenesis is directly linked with changed expression of claudins. It is believed that expression of claudins might be regulated on many levels: transcriptional, posttranscriptional, or recently suggested epigenetic regulations (miRNA, DNA methylation, and histone modifications) take place [17]. In neoplastic tissues, the expression of 14 claudins was found both down- and upregulated. Claudin-3 and claudin-4 are the most frequently deregulated in cancers and are found highly expressed in ovarian, breast, and prostate cancers. It also seems that the changes in the expression of claudins are more sophisticated; for example, claudin-1 was reported to be downregulated in breast cancer, but other studies found it to be upregulated. The differences might be associated with different types of tumors, its direct localization, invasiveness, as well as stage [17]. On the other hand, the discrepancies in these results indicate the importance of understanding the role of claudins in basic physiological as well as pathophysiological processes in humans.

## 2. Claudins in Fertility

The basic function of the reproductive system is to enable fertilization. Both ovaries and testis play a crucial role in the production of hormones and maintaining fertility [18].

### 2.1. Testis

**Figure 2:**
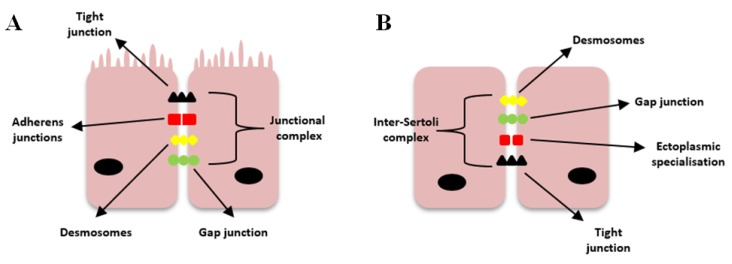
Diagram illustrating the difference between epithelial cells (**A**) and seminiferous epithelia (**B**). Tight Junctions (TJs) between Sertoli cells are located in the basal part of the cells, while in other epithelial cells, they are located in the apical part. Between Sertoli cells occur “ectoplasmic specialization”, which is a testis-specific junction.

The blood–testis barrier (BTB) is a physical barrier between the testicles and blood vessels. It consists of capillary endothelium, testicular seminiferous membrane, and intercellular connections of Sertoli cells. This structure limits the flow of substances from the blood to the testis, which protects sperm-forming cells against antibodies and antigens (contact could damage them and, as a consequence, lead to infertility). TJs play a pivotal role in this process and form together with other molecules, a large complex called inter-Sertoli cell junctional complex [19] (Figure 2).

Claudin-11 is reported to participate in the proper functioning of Sertoli cells and has been linked with men’s fertility. Studies in mice showed that silencing of claudin-11 results in loss of epithelial phenotype in Sertoli cells [20]. The increase in the expression of claudin-11 and its dislocation is associated with impaired spermatogenesis [21,22,23,24]. Moreover, McCabe et al. demonstrated that chronic gonadotropin suppression affects the expression of claudin-11 in BTB, which leads to a reduction in the number of germ cells from the seminal epithelium, crucial for male hormonal regulation [25]. Another factor that might affect claudin-11 expression is mirabegron, a drug used to treat urinary incontinence. In rat Sertoli cells, mirabegron increases claudin-11 expression through the p44/42 MAPK signaling pathway and changes the localization of this protein [26]. Pan et al. investigated the effect of varicocele (VC) on the blood–testis barrier in rat testicles and found claudin-11 expression significantly reduced [27]. In response to scrotal heat stress, the expression of claudin-11 was also changed, but in contrast to the previous study, its expression was elevated [28]. Testosterone and 5-α-dihydrotestosterone (DHT) might also affect claudin-11 mRNA expression. Moreover, this process was inhibited by non-steroidal androgen receptor antagonist (flutamide), which suggests that the increase in claudin-11 expression is androgen-dependent [29]. Similar findings were reported by Florin et al. [30]. Dehydroepiandrosterone sulfate (DHEAS) is a metabolite of dehydroepiandrosterone (DHEA) which circulates in bloodstream at a higher concentrations. Recent studies showed that DHEAS increases expression of claudin-3 and claudin-5 in murine Sertoli cell line TM4 via induction of Erk1/2 kinase phosphorylation and transcription factors cAMP response element-binding protein (CREB) and activating transcription factor 1 (ATF-1) [31].

### 2.2. Uterus

Studies in rat vagina showed that expression of claudins is modulated by estrogen loss of this hormone, which leads to the downregulation of the expression of TJs, which in consequence suggests that estrogen might modulate epithelial permeability [32]. The expression of claudins is also reported to be present in endometrium, and their expression is different and depends on the day of the menstrual cycle. Claudin-4 expression is reported to be enhanced in the luteal phase of the menstrual cycle [33]. A close interaction between embryo and endometrium is necessary for proper implantation and is associated with numerous structural and morphological changes, among others, in TJs [34]. Claudin-4 expression in the uterine epithelium increases during implantation and is regulated by ovarian hormones [35]. Interestingly, in a group of women with idiopathic infertility and with eutopic endometrium, the expression of claudin-4 was upregulated as compared to the control (fertile) group [36]. On the other hand, a decreased expression of claudin-3 and claudin-4 is associated with endometriosis [37], which is known for its significant contribution to the reduction of fertility. Claudin-7 is also reported to participate in fertilization. Its expression was examined in the pregnant rats and showed that during implantation, expression of claudin-7 significantly decreases as compared to the first day of pregnancy; moreover, its location also changes [38]. Other studies in rats showed that biseptol-A, which is used to treat respiratory, urinary, and gastrointestinal infections in the perinatal period and early pregnancy, affects the expression of claudins in the uterus of the offspring, which changes the susceptibility of the uterus to implantation, and, in consequence, reduces fertility [39]. Similarly to the endometrium, the expression of claudins was also reported in the luminal epithelial surface of the fallopian tube [40]. One of the consequences of a disturbance in the structure of epithelial cells in the fallopian tube is ectopic pregnancies (EC). Zhaeentan et al. demonstrated that hydrocortisone regulates the expression of claudin-3 and claudin-4, of which increasing expression contributes to strengthening intercellular TJs in the fallopian tube [41]. Claudins might also participate in the proper functioning of the placenta. In the middle of mice pregnancy, the placenta showed increased expression of claudins 1–7, claudin-11, claudin-12, and claudin-23 (especially claudin-1, -2, -4, and -5), indicating their role in paracellular transportation during exchange of, e.g., nutrients, ions between mother and fetus. Moreover, their expression was reported to be modulated by steroid hormones [42]. Amniotic fluid volume is regulated by absorption across the amniotic epithelial cells [43]. Kobayashi et al. investigated the expression of claudin-4 and claudin-7 in human amnion during late pregnancy [44]. Their expression decreased during pregnancy progression, as well as after decreasing inflammation in the cell culture model treated with dexamethasone [44].

## 3. Claudins in Human Cancer

**Figure 3:**
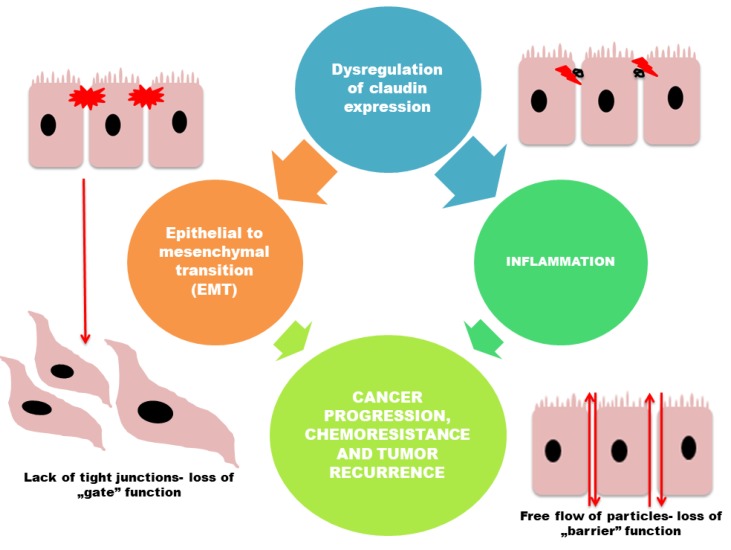
Diagram showing the involvement of claudins in cancer initiation and progression.

Cancer is one of the leading causes of death worldwide. It includes a set of diseases that differ in location, while their common features are uncontrolled cell proliferation, loss of controlled cell death, invasiveness, and metastasis [45]. Its occurrence is a multistep process in which many both genetic and environmental factors are included. The lifestyle, physical activity, metabolic disorders associated with diet as well as environmental pollutants are also causative factors in the process of carcinogenesis [46]. Neoplasm, which originates from epithelial tissue, is the most common type of malignancies. Epithelial–mesenchymal transition (EMT) is a complex process in which epithelial cells transform into mesenchymal cells under the influence of various factors. During EMT in epithelial cells, the loss of junction and correct polarity, remodeling of the cytoskeleton, reprogramming of gene expression, and, in consequence, a change in signaling pathway which enables metastases and invasiveness of cancer cells takes place [47,48,49]. Thus, loss of the expression of claudins and, in consequence, remodeling of TJs stimulates EMT and cancer metastases. In this case, the role of claudin-1 is well documented; a decrease in the expression is directly associated with the EMT process [50]. However, the associations are not always linear, like in case of claudin-3 or claudin-4 in ovarian cancer, where its expression is increasing during metastases [51]. The incidence of cancer is also strongly associated with chronic inflammation [46]. Activation of inflammatory response increases cell proliferation, tumor growth, invasiveness, and modulates the response to anticancer drugs. TJs play the role of the so-called “barrier” that provides adequate permeability to various molecules. Modulation of the expression of claudins leads to the change in the levels of inflammatory cytokines [1,52], which, in consequence, induces tumor growth and progression. For example, TNF-α affects the expression of claudin-1 in colorectal cancer and thus induces EMT [50]. In summary, impaired expression of claudins causes disorganization of TJs, which, in consequence, leads to malfunction of epithelial cells associated with aggressive phenotypes in cancer (Figure 3). However, it is worth emphasizing that claudins may act as tumor promoters or suppressors, dependent on the tissue type, progression of the disease, or hormonal status [2,6].

### 3.1. Claudins in Endocrine-Related Cancers

Both cancer incidence and mortality are growing worldwide and are predicted to be higher with a growing population, longer lifespan time, civilization, and environmental risk factors. One from five men and one from six women will develop cancer during their lifetime [53]. Endocrine-related cancers of the reproductive system are one of the commonest cancers worldwide. Breast cancer constitutes the most common cancer among women, whereas cervical cancer is the fourth one [54]. Prostate cancer is the second most common cancer in men worldwide, whereas, in the United States of America, is the most common [45]. It is one of the most commonly diagnosed cancer in half of the world’s countries. In the case of breast, cervical, and prostate cancers, the mortality is decreasing with years, but the incidence is estimated to be growing [55]. Recent research indicates that both high expression of claudins, as well as its lack of expression, might serve as a marker of the process of tumor initiation and progression (Table 2).

### 3.2. Localization of Claudins in Cells

Claudins mostly occur in the apical part of the cell, but they might also be present in the nucleus and cytoplasm, and their reorganization in cells might influence cell behavior, e.g., relocation of claudins is observed in cancer. Dhawan et al. showed that the expression of claudin-1 is not only upregulated in human primary colon cancer, colon cancer-derived liver metastases, and lymph nodes metastases, but also relocated. Instead of membrane localization, claudin-1 was localized in the nucleus and cytoplasm [80]. Its delocalization into the nucleus was also observed in human osteosarcoma cells [81] and follicular thyroid carcinoma cells [82]. Moreover, claudin-2 was observed in the nucleus in human lung adenocarcinoma cells [83], and claudin-3 was reported to be present in nuclei in breast cancer cell lines [84] and human colorectal cancer [85]. Changes in claudin-4 location in endometrial cancer cells were also observed [86]. Interestingly, relocation of claudin-1 is not associated with protein modification, based on not-observed changes in the mass of this protein [80], whereas claudin-2 was reported to be dephosphorylated when located in nuclei of cells [83]. Changed localization of claudin-11 from membrane to cytoplasm was observed in Sertoli cells in infertile men [23]. It is generally believed that localization of claudins in nuclei of cancer cells is associated with cell signaling modulation and regulation of gene transcription; however, this statement is not confirmed so far. It was suggested that protein kinases A and C (PKA, PKC, respectively) activate strong nuclear localization signals (NLS) to exchange the cytoplasmic fraction of claudin-1 to nuclear fraction [82]; however, not all claudins present strong NLS sequence necessary for activation. Nevertheless, it is obvious that changes in claudins localization in cells are directly associated with their role in carcinogenesis.

### 3.3. Hormonal Regulation of Claudins

The growing body of evidence suggests that the expression of claudins might be regulated hormonally. Cuevas et al. showed that claudin-4 expression in HEC-1A cells exposed to physiological concentrations of estradiol (100 nM) was modulated, and its localization was changed. Cells without hormonal treatment presented most of the claudin-4 localization in cytoplasm and membrane, whereas 50 nM of estradiol caused an increase in the expression and membrane localization [86]. Claudin-11 is known to be regulated hormonally, and its knockout models result in infertility [87]. In rat primary cultures of testicular Sertoli cells, claudin-11 expression was reported to be modulated both by testosterone as well as follicle-stimulating hormone (FSH) [88]. Additionally, in mice models, suppressed levels of gonadotropins affected the localization of claudin-11 in Sertoli cells [25]. Similarly, claudin-3 and claudin-5 were reported to be regulated by dehydroepiandrosterone sulfate (DHEAS)- one of the most abundant steroids in humans. Phosphorylation of Erk1/2 and transcription factors CREB and ATF-1 in a murine in vitro model caused increased expression of claudin-3 and claudin-5 [31]. Claudin-5 was also reported to be regulated by estrogen in vascular endothelium, where its expression was significantly increased after estradiol treatment, indicating its role in vascular integrity [89]. In amniotic epithelia and placenta, claudin-3 and claudin-4 expression were increased by progesterone in a dose-dependent manner, but interestingly without their delocalization [44]. Similarly, claudin-7 expression, which naturally changes during pregnancy, was also reported to be modulated in response to estrogen and progesterone changes [38]. The expression of claudin-1 in breast cancer seems to be also modulated by a hormonal response. Treatment of Michigan Cancer Foundation-7 (MCF-7) breast cancer cells with tamoxifen, a well-known anti-estrogen drug, increased claudin-1 expression [90]. Aravindakshan et al. showed that in normal murine epithelial cells in ovary expression of claudin-3, claudin-4, and claudin-11 is affected by FSH, luteinizing hormone (LH), estradiol, and testosterone. Interestingly, they did not observe a hormonal regulation of claudin-1 expression in these cells [91]. Thus, it might be concluded that, in both physiological as well as pathological conditions, claudins are hormonally-regulated proteins.

### 3.4. Claudins in Prostate Cancer

The prostate gland is a part of the male genitourinary system playing a critical role in the functioning of the reproductive system. The fertility of men depends directly on the content of prostate fluid secreted by the epithelium of the prostate, which constitutes from one-fifth to one-third of ejaculate. The prostate, as hormone-dependent organ, is changing with aging, the ratio of estrogens to androgens is increasing, and the production of growth factors and other hormones modulates the proliferation of prostatic cells [45].

The direct cause of prostate cancer is still unknown. It is believed that age, ethnicity, genetic predisposition, diet, and lifestyle might act as causative factors in initiation and progression [92]. A milestone has been made in screening test of the disease; mainly by the monitoring of prostate-specific antigen (PSA), the lethality of prostate cancer is decreasing, although the incidence is still growing [45]. The begging of metastatic cascade starts with the loss of cell–cell adhesion, which enables migration. Moreover, chronic inflammation in the prostate is believed to participate in tumorigenesis [93]. Thus, the interest in the role of claudins in prostate cancer is huge. The expression of claudin-3 and claudin-4 correlates with advanced prostate tumor stage and its recurrence [51,66]. Studies on the expression of claudin-3 in various type of prostate disease—prostatic adenocarcinoma (NAC), benign prostatic hyperplasia (BPH), prostatic intraepithelial neoplasia (PIN), prostate cancer, and metastatic prostatic adenocarcinoma (Mets)—reported that expression of theses claudins was upregulated in malignant tumor [66]. Väre et al. reported that expression of claudin-3 and claudin-4 was elevated in prostate adenocarcinoma. The same researchers noticed that expression of claudin-1, claudin-2, and claudin-5 was lower in adenocarcinoma cells as compared to healthy prostate tissue [55]. Ye et al. also observed that the expression of claudin-3 in prostate cancer is increased [67]. Therefore, it seems that claudin-3 might act as a biomarker of prostate cancer to be possibly used in the early detection of neoplasm. Moreover, claudin-3 and claudin-4 have become promising candidates in the new treatment strategy against prostate cancer. *Clostridium perfingens* enterotoxin (CPE) is a polypeptide that binds to claudin-3 and claudin-4 receptors, causing cell death by the influx of calcium ions into the cell [94,95]. The usefulness of this fact might allow effective treatment of androgen-resistant patients, but further research seems necessary to check the effects of CPE on other tissues that also express these claudins. Romanov et al. constructed a protoxin that selectively attaches only to cells that express claudin-3, claudin-4, and PSA [96]. It significantly increases the possible usefulness in therapy by reduction of the cytotoxicity of enterotoxin. The expression of claudin-8 is significantly increased as compared to BPH. Moreover, research indicates that claudin-8 promotes proliferation and migration of prostate cancer cells [78]. Won Seo et al. noticed that the low expression of claudin-1 and claudin-5 is associated with a high Gleason score, indicating that low expression of these claudins is associated with the malignancy [56,97]. Previous studies also noticed the relationship between the expression of claudins and the Gleason score: Väre et al. reported that low expression of claudins-1, -4, and -5 is associated with the Gleason score and it affects patient prognosis [55]. The changing concentration of androgens might also modulate the expression of claudins: low serum testosterone causes disorganization of TJs ultrastructure by reduced protein levels of claudin-4 and claudin-8 [98]. Both the expression of claudin-2 and claudin-7 was reported to be downregulated in prostate cancers [63].

### 3.5. Claudins in Breast Cancer

It was estimated that during 2018, two billion women would be diagnosed with breast cancer [99]. Still, breast cancer constitutes the most common cancer in women. The need for new therapeutic strategies besides surgical intervention, chemotherapy, and radiotherapy is still highlighted. The etiology of the disease is complex and involved genetic mutations, family history, and environmental pollutants that might disturb the hormonal balance in women [100]. The tumors display both inter- and intra-tumoral heterogeneity. Thus, the need for uncovering the subtypes of breast cancer is crucial in understanding the cellular mechanism of tumorigenesis [101]. The method of treating breast cancer depends on its subtype. Claudins expression might be different in different subtypes of breast cancer, as presented in Table 2. Moreover, a claudin-low molecular subtype of breast cancer has been distinguished. It is characterized by low or not-present expression of luminal markers, decreased expression of claudin-3, claudin-4, and claudin-7 [17,68], high expression of EMT and immune genes. The majority of claudin-low cancers are triple-negative and are believed to originate from mammary epithelial stem cells [102]. Carcinoma of the breast develops locally and metastasizes to lymph nodes and internal organs, mainly to the liver [64]. Therefore, the expression of claudins was evaluated for possible usage as a biomarker in predicting liver metastases. Kimbung et al. noticed that claudin-2 may not only be a marker of tumor recurrence but may also serve as a marker of potential liver metastasis [64]. An application of claudin-2 as a biomarker would allow for a more personalized approach to the patient and better treatment results. The expression of claudin-1 was reported to be downregulated in invasive breast carcinoma [57]. The contradictory effect was observed in the basal type of high-grade invasive ductal breast cancer [59]. A similar contradictory effect was observed for claudin-4 and claudin-7. The expression of claudin-4 is upregulated in the basal type of high grade [71], whereas downregulated in invasive ductal grade 1 [57]. Claudin-7 expression is decreased in invasive ductal carcinoma [76] and increased in luminal type [68].

A lot of research has shown that the expression profile of claudins is different in various breast cancer tissues and affects the response to chemotherapy. Tamoxifen increased claudin-1 expression, which has an anti-apoptotic effect in human hormone-dependent MCF-7 cells but not in T-47D cells [90]. This study suggests that evaluation of the profile of the expression of claudins before tamoxifen therapy might provide better treatment results. Moreover, the dysregulation of claudin-1 provides EMT. Blanchard et al. showed that expression of claudin-1 in breast cancer cell line MCF7 is upregulated by protein kinase C (PKC), which is necessary for activation of the extracellular-signal-regulated kinase (ERK) signaling pathway [103]. The evaluation of claudins expression was also suggested to increase the possibility of creating new drugs. Iravani and co-workers showed that expression of claudin-12 is upregulated in estrogen receptor (ER)-negative breast cancer and its expression is associated with poor prognosis, indicating that claudin-12 may serve as a marker for predicting the survival in this cancer subtype [79]. The overexpression of claudin-6 was showed to inhibit migration and invasion of MCF-7 cells [104]. Lu et al. demonstrated that SMAD2 causes downregulation of claudin-6 by DNA (cytosine-5)-methyltransferase 1 (DNMT1)-mediated DNA methylation, which results in increased migration and invasion of breast cancer cells [105]. In addition, research has shown that estrogen receptor β (ERβ) plays an anti-cancer role in breast cancer, and Song et al. demonstrated that this effect is mediated by claudin-6 [106]. On the other hand, Yang et al. demonstrated that claudin-6 affects chemo-resistance in MDA-MB-231 cells in response to adriamycin (ADM) by reducing the drug-induced apoptosis [107]. Previous studies reported that decreased expression of claudin-2 is associated with increasing stage of cancer and with lymph node metastasis [108,109]. Tabariès and co-workers showed that afadin, an actin filament-binding protein, might create a complex with claudin-2 that enables the efficient formation of metastases. The results revealed the possibility of using a combination of claudin-2 and afadin as prognostic markers that may allow better detection of metastatic potential [110].

### 3.6. Claudins in Cervical Cancer

Cervical cancer is the fourth most common cancer among women. About 570,000 new cases are estimated to be diagnosed each year [111]. It is also the most common gynecological cancer diagnosed in pregnancy [112]. Two major types of cervical cancer might be distinguished: squamous cell carcinoma and adenocarcinoma, of which the first one occurs more often. Human papilloma virus (HPV) infection is responsible for most of the new cervical cancer incidence, especially in countries where the screening program and vaccination are not present [111]. Although early-stage and locally-advanced cervical cancers are well cured, patients diagnosed with metastatic and recurrent cervical cancer have poor and limited treatment options [113]. Thus, understanding the molecular events associated with cervical carcinogenesis might benefit new treatment strategies. Claudin-1 has been proposed as a biomarker of cervical histology and cytology. Increased expression of claudin-1 in cervical cancer was noticed by Zhang et al. [60] and Hoellen et al. [61]. Significant overexpression of claudin-1 was observed in tumor cells; although no correlation was found with Federation of Gynecology and Obstetrics (FIGO) stage, claudin-1-postive patients exhibited more lymph node metastasis as compared to claudin-1 negative patients [61]. A lot of studies have reported that the expression of claudin-1 correlates with tumor invasion: it is said that claudin-1 may improve anti-apoptosis ability and stimulate metastasis [60]. Moreover, Márta et al. suggested that claudin-1 may be useful as a diagnostic marker in cervical cancer, just as good as the existing biomarker, p16^INK4a^ [114]. p16^INK4a^ is a protein that plays an important role in the cell cycle in cervical cancer; when cell cycle control is impaired and its expression increases. Researchers compared claudin-1/Ki67 double immunoreaction with a commercially available test, p16^INK4a^/Ki67. Sensitivity and specificity were higher in the claudin-1/Ki67 test, but it was not statistically significant (sensitivity 74.00% vs. 76.00%, specificity 81.38% vs. 85.67%) [115]. Zhu et al. noticed that the expression of claudin-5 and claudin-9 is downregulated in cervical carcinoma tissues compared with adjacent non-neoplastic tissues, while claudin-8 is elevated [74]. In contrast to claudin-1, claudin-6 is downregulated in cervical carcinoma, and expression of claudin-6 leads to decreased cell proliferation, colony formation in vitro, and tumor growth in vivo [116]. Based on this observation, it was suggested that this protein may be a tumor suppressor for cervical cancer. This is confirmed by the result of Zhang et al., who noticed that expression of claudin-6 is associated with apoptosis signal-regulating kinase 1 (ASK1) [75].

### 3.7. Claudins in Ovarian Cancer

Ovarian cancer is the seventh most common cancer worldwide and is the leading cause of cancer-associated death in highly developed countries in women [117]. This is due to the fact that it is diagnosed at an advanced stage, with of lack of early symptoms. Standard therapy of newly diagnosed ovarian cancer is based on cytoreductive surgery and platinum-based chemotherapy. Most women respond well to chemotherapy; however, there is a high rate of recurrence in the first two years [54]. Thus, the need for novel treatment options is highlighted. One of the stages in the diagnosis of ovarian cancer is testing the concentration of molecular markers in blood serum. Currently, the OVA1 test is available and it checks five markers: transthyretin, apolipoprotein A-1, microglobulin β2, transferrin, and CA-125. This test is very useful, but its specificity and sensitivity are limited. Claudins possess the potential to serve as new molecular markers of ovarian cancer. Previous research showed that claudin-3 is overexpressed in about 90% of ovarian tumors. Huang et al., by using *CLDN3* - gene silencing, proved that cell proliferation and tumor growth were reduced and the number of apoptotic cells increased significantly [70], indicating that this protein may be a new target in ovarian cancer treatment, which confirms previous reports [69]. Similarly to claudin-3, claudin-4 is highly elevated in ovarian cancer [69]. Expression of this protein affects apoptosis resistance and migration of cancer cells [118]. The expression of claudin-4 also affects treatment results—it reduces cell apoptosis in response to paclitaxel [119]. Yoshida et al. suggested that claudin-4 might contribute to platinum resistance in ovarian cancer [120], but current studies did not show a confident association between claudin-4 expression and platinum response [121], so this issue remains unclear. Interestingly, in addition to claudin-3 and claudin-4, claudin-6 might serve as a novel receptor for *Clostridium perfinges* enterotoxin in ovarian cancer [122]. Dahiya et al. investigated the relationship of claudin-7 with ovarian neoplasm [77]. They noticed that claudin-7 is upregulated in most of the cases of ovarian cancer. Moreover, they proved that the increased expression of claudin-7 affects the invasion of ovarian cancer cells. Research conducted in the last few years has shown that elevated expression of claudin-7 is associated with poor overall survival in people with ovarian serous carcinoma [123]. It is worth emphasizing that high expression of claudin-7 in ovarian carcinoma cells causes reduced response to first-line platinum-based combination chemotherapy, and silencing of its expression leads to increased sensitivity to cisplatin [124], confirming its involvement in the response to chemotherapy.

### 3.8. Claudins in Endometrial Cancer

Endometrial cancer is the most common malignant cancer of women’s reproductive tract [117]. It can be diagnosed and treated in early stages due to typical symptoms [125]. Histopathology examination is a gold standard in the diagnosis of endometrial cancer, but due to its invasive nature, it cannot serve as a clinical monitoring method [125]. Based on this, new strategies are needed. Previous studies that examined the expression of claudin-2 and claudin-3 during the transformation from normal tissue to endometrioid adenocarcinoma showed that their expression increases with the progression of the tumor, suggesting that these proteins could serve as good biomarkers for early diagnosis of premalignant lesions [65]. In contrast to breast cancer, claudin-6 in endometrial cancer possesses a different function. Cao et al. demonstrated that knockdown of *CLDN6* provides decreased cell proliferation and migration, which might be beneficial in targeted therapies [126]. The expression of claudin-7 and its function in endometrial cancer cells (AN3 CA cell line) were investigated by Li et al. [127]. Researchers demonstrated that claudin-7 plays an important role in endometrial cancer; reduction of the expression of claudin-7 caused increased cell proliferation and invasiveness. A change in claudin-1 and claudin- 4 expression is also associated with endometrial cancer. Zhao et al. demonstrated that the expression of these proteins is different in distinct endometrium tissues [62]. Moreover, the expression of claudin-4 is upregulated, while claudin-1 is downregulated as compared to normal endometrial cells [62], indicating that these proteins might be involved in endometrial tumorigenesis and could be helpful during the diagnosis of endometrial cancer. This hypothesis concerning claudin-4 was also confirmed by the study carried out by Pan et al. [128]. In addition, Kölbl et al. used claudin-4 with other markers to detect circulating tumor cells (CTCs) [72]. The results showed that claudin-4 expression is increased in most cell lines used in that study, indicating that it might serve as a good marker for detecting CTCs [72]. On the other hand, Tessier-Cloutier et al. showed that in differentiated and undifferentiated endometrial cancers, claudin-4 expression is absent or very low [73]. Due to the fact that estrogen may affect the development of endometrial cancer, sex hormones might play an important role in the regulation of TJs in normal and cancer cells. Someya et al. showed that estradiol increases the expression of claudin-3 and claudin-4, while progesterone inhibits this effect in the uterine cancer Sawano cell line. Interestingly, in human endometrial epithelial cells, the effect of estradiol and progesterone was contradictory: progesterone induced expression of claudins, whereas estradiol had no effect [129]. The results of this study indicate that the expression of claudins is naturally regulated during hormonal changes in women’s endometriums.

## 4. Conclusions

It is generally believed that tumorigenesis is associated with loss of TJs and decreased expression of claudins. However, the results from recent years have showed that the expression of claudins is more complex and dependent on the type of tissue, the progression of the disease, a subtype of cancer, or its origin. Nevertheless, claudins might serve as potential biomarkers of disease and its progression, especially in cancers of the human reproductive system, which constitutes the most common cancers worldwide. Notably, more research studies concerning the role of claudins in the chemo-resistance of cancer cells might bring a new light of their importance. Furthermore, a better understanding of the role of claudins in reproductive tissues could help increase the knowledge of the causes of infertility in patients with idiopathic infertility and thus help in choosing the right assisted reproductive technique that will bring better therapeutic effects.

## Figures and Tables

**Table 1:** Division of claudins based on the sequence similarity and their chromosomal localization, protein size, and molecular weight.

Gene	Localization	Protein Size (AA)	Molecular Weight (kDa)
**Classic Claudins**			
*CLDN1*	3q28	211	22.74
*CLDN2*	Xq22.3	230	24.55
*CLDN3*	7q11.23	220	23.32
*CLDN4*	7q11.23	209	22.08
*CLDN5*	22q11.21	218	23.15
*CLDN6*	16p13.3	220	23.29
*CLDN7*	17p13	211	22.39
*CLDN8*	21q22.11	225	24.85
*CLDN9*	16p13.3	217	22.85
*CLDN10*	13q32.1	226	24.25
*CLDN14*	21q22.3	239	25.70
*CLDN15*	7q11.22	228	24.36
*CLDN17*	21q22.11	224	24.60
*CLDN19*	1p34.2	224	23.23
**Non-Classic Claudins**			
*CLDN11*	3q26.2	207	21.99
*CLDN12*	7q21	244	27.11
CLDN13*			
*CLDN16*	3q28	305	33.84
*CLDN18*	3q22.3	261	27.86
*CLDN20*	6q25	219	23.52
*CLDN21*	4q35.1	NI	NI
*CLDN22*	4q35.1	220	24.51
*CLDN23*	8p23.1	292	31.92
*CLDN24*	4q35.1	205	22.80

* absent in humans and chimpanzees, NI—no information; *CLDN*—claudin gene; AA—amino acids.

**Table 2:** Summarized changes in claudin expression in hormone-related cancers in the human reproductive system.

Claudin	Cancer	Changing Expression	Reference
Claudin-1	Prostate	↓	[55,56]
	Breast (Invasive ductal carcinoma)	↓	[57]
	Breast (Luminal A and B)	↓	[58]
	Breast (Triple-negative/basal-like)	↓	[58]
	Breast (HER2 positive)	↑	[59]
	Breast (Claudin-low)	↓	[59]
	Cervical	↑	[60,61]
	Endometrial	↓	[62]
Claudin-2	Prostate	↓	[55,63]
	Breast cancer metastases	↑	[64]
	Endometrial	↑	[65]
Claudin-3	Prostate	↑	[51,55,66,67]
	Breast	↓	[17,68]
	Ovarian	↑	[69,70]
	Endometrial	↑	[65]
Claudin-4	Prostate	↑	[51,55,66]
	Breast (Invasive ductal carcinoma)	↓	[17,57,68]
	Breast (basal-like)	↑	[71]
	Ovarian	↑	[69]
	Endometrial	↑	[62,72]
	Endometrial (differentiated and undifferentiated)	↓	[73]
Claudin-5	Prostate	↓	[55,56]
	Cervical	↓	[74]
Claudin-6	Cervical	↓	[75]
Claudin-7	Prostate	↓	[63]
	Breast (Invasive ductal carcinoma)	↓	[17,59,68,76]
	Breast (luminal type)	↑	[68]
	Ovarian	↑	[77]
Claudin-8	Prostate	↑	[78]
	Cervical	↑	[74]
Claudin-9	Cervical	↓	[74]
Claudin-12	Breast (ER-negative)	↑	[79]

HER2—receptor tyrosine-protein kinase erbB-2; ER—estrogen receptor; ↓downregulated; ↑upregulated.
